# FOXO target gene CTDSP2 regulates cell cycle progression through Ras and p21^Cip1/Waf1^

**DOI:** 10.1042/BJ20140831

**Published:** 2015-07-06

**Authors:** David E.A. Kloet, Paulien E. Polderman, Astrid Eijkelenboom, Lydia M. Smits, Miranda H. vanTriest, Maaike C.W. vandenBerg, Marian J. Groot Koerkamp, Dik vanLeenen, Philip Lijnzaad, Frank C. Holstege, Boudewijn M.T. Burgering

**Affiliations:** * University Medical Centre Utrecht, Universiteitsweg 100 STR3.217, 3584CG Utrecht, The Netherlands

**Keywords:** cell cycle progression regulation, C-terminal domain small phosphatase 2 (CTDSP2), forkhead box O (FOXO), gene expression regulation, growth factor signalling, p21^Cip1/Waf1^

## Abstract

Activity of FOXO (forkhead box O) transcription factors is inhibited by growth factor–PI3K (phosphoinositide 3-kinase)–PKB (protein kinase B)/Akt signalling to control a variety of cellular processes including cell cycle progression. Through comparative analysis of a number of microarray datasets we identified a set of genes commonly regulated by FOXO proteins and PI3K–PKB/Akt, which includes CTDSP2 (C-terminal domain small phosphatase 2). We validated CTDSP2 as a genuine FOXO target gene and show that ectopic CTDSP2 can induce cell cycle arrest. We analysed transcriptional regulation after CTDSP2 expression and identified extensive regulation of genes involved in cell cycle progression, which depends on the phosphatase activity of CTDSP2. The most notably regulated gene is the CDK (cyclin-dependent kinase) inhibitor p21^Cip1/Waf1^ and in the present study we show that p21^Cip1/Waf1^ is partially responsible for the cell cycle arrest through decreasing cyclin–CDK activity. Our data suggest that CTDSP2 induces p21^Cip1/Waf1^ through increasing the activity of Ras. As has been described previously, Ras induces p21^Cip1/Waf1^ through p53-dependent and p53-independent pathways and indeed both p53 and MEK inhibition can mitigate the CTDSP2-induced p21^Cip1/Waf1^ mRNA up-regulation. In support of Ras activation by CTDSP2, depletion of endogenous CTDSP2 results in reduced Ras activity and thus CTDSP2 seems to be part of a larger set of genes regulated by FOXO proteins, which increase growth factor signalling upon FOXO activation.

## Abstract (precis)

Growth factor controlled activity of forkhead box O transcription factors results in altered gene expression, including expression of CTDSP2 (C-terminal domain small phosphatase 2). CTDSP2 can regulate cell cycle progression through Ras and the cyclin-dependent kinase inhibitor p21^Cip1/Waf1^.

## INTRODUCTION

The forkhead box transcription factors are a large family of transcription factors characterized by a conserved DNA-binding domain: the forkhead box. The O-group of forkhead box factors consists of four members in mammals; FOXO1, FOXO3, FOXO4 and FOXO6. Growth factor–PI3K (phosphoinositide 3-kinase)–PKB (protein kinase B)/Akt signalling is a major regulator of FOXO activity. FOXO proteins are direct substrates of PKB/Akt and are phosphorylated on conserved residues, resulting in their inactivation. On the other hand, under conditions of cellular stress, i.e. following an increase in reactive oxygen species, FOXO proteins are activated, which involves JNK (c-Jun N-terminal kinase) and ubiquitination of FOXO proteins (reviewed in [1]). FOXO proteins have been implicated in the regulation of a large number of processes (reviewed in [2]). FOXO signalling is evolutionarily conserved and, in the nematode *Caenorhabditis elegans*, growth factor–PI3K–PKB/Akt signalling regulates the FOXO orthologue DAF-16. Enhanced DAF-16 activity due to reduced insulin signalling increases longevity of *C. elegans* and similarly in *Drosophila melanogaster* dFOXO activity affects lifespan. In humans, SNPs (single nucleotide polymorphisms) have been identified in FOXO3 that are associated with increased lifespan. These and other results suggest that a FOXO-induced gene expression programme affects longevity, but at present it remains elusive as to which FOXO target genes convey this organism-wide effect. To identify genes transcriptionally controlled by FOXO, which are critical in mediating the FOXO proteins’ effect on lifespan, a number of laboratories have used microarrays to explore mRNA changes after FOXO activation (the present study and [3–6]). These studies show that a large part of FOXO transcriptional output is highly context-dependent and FOXO regulation of most genes is only observed in a limited number of settings or cell types. CTDSP2 (C-terminal domain small phosphatase 2), also referred to as SCP2 or OS4, is regulated in all datasets that we analysed, but has not been described as a FOXO target gene previously. CTDSP1, CTDSP2, CTDSPL (CTDSP-like) and CTDSPL2 are phosphatases and related to CTDP1 (FCP1 in yeast) because of their characteristic phosphatase domain [7]. Similar to CTDP1, CTDSP family members have been shown to dephosphorylate the CTD (C-terminal domain) of RNAPII (RNA polymerase II) core subunit RBP1 [7,8] and thereby inhibit gene expression [8–10]. Other studies have highlighted different roles of CTDSP1, CTDSP2 and CTDSPL, including regulation of TGFβ (transforming growth factor β) signalling [11–14], Snail protein stability [15] and cell cycle progression [16,17]. In conclusion, members of the CTDSP family of phosphatases are involved in regulation of both signalling and transcription.

In the present study, we show that CTDSP2 is consistently regulated in a range of microarray datasets generated from cell lines overexpressing FOXO3 or FOXO4. We find that CTDSP2 is a direct target gene of FOXO proteins with FOXO-binding sites directly adjacent to the TSS (transcriptional start site) of CTDSP2, which are sufficient for transactivation. We show that CTDSP2 is regulated by FOXO1, FOXO3 and FOXO4 and its expression is highly sensitive to PI3K–PKB/Akt–FOXO signalling. One of the consequences of ectopic expression of CTDSP2 is a strong reduction of the number of S-phase cells. However, unlike previous suggestions [16,17], we do not confirm the requirement of the pocket protein Rb (retinoblastoma) for this. Instead, microarray analysis of cells expressing CTDSP2 reveals several genes that are regulated, which in turn are potentially involved in regulating S-phase onset. Of these, we show that the CDK (cyclin-dependent kinase) inhibitor p21^Cip1/Waf1^ contributes significantly to the decreased cell cycle progression of CTDSP2-overexpressing cells. Our data indicate that p21^Cip1/Waf1^ is up-regulated in response to activation of Ras by CTDSP2. Indeed, depletion of endogenous CTDSP2 results in decreased activity of Ras, as well as PKB/Akt. Interestingly, activation of both Ras and PKB/Akt is a known effect of FOXO activation and this appears to involve CTDSP2.

## MATERIALS AND METHODS

### Tissue culture

HEK (human embryonic kidney)-293T (ATCC CRL-11268), NIH/3T3 (ATCC CRL-1658) and U2OS (ATCC HTB-96) cells, wild-type MEFs (mouse embryonic fibroblasts), p107/p110/p130-deleted MEFs [18] and all derived lines were maintained in DMEM (Dulbecco's modified Eagle's medium) with 10% (v/v) FBS, L-glutamine and penicillin/streptomycin. RPE (retinal pigment epithelium) (ATCC CRL-4000) cells were maintained in DF12 with 10% (v/v) FBS, L-glutamine and penicillin/streptomycin. DLD1 (ATCC CCL-221) and derived DL23 [19] cells were maintained in RPMI 1640 medium with 10% (v/v) FBS, L-glutamine and penicillin/streptomycin.

### Antibodies and reagents

Anti-CTDSP2 antibodies were purchased from Abcam [mAb (monoclonal antibody) 2230C1a and pAb (polyclonal antibody) ab97463]. Anti-TUBA (α-tubulin) was purchased from Calbiochem. Anti-p21^Cip1/Waf1^ was purchased from BD Biosciences. Anti-p53 (DO-1), anti-cyclin E (C-19), anti-cyclin E (HE-12), anti-CDK2 (M-2), anti-CDK6 (M-21), anti-N-ras (SC-31) and anti-ERK1 (extracellular-signal-regulated kinase 1) (K-23) were purchased from Santa Cruz Biotechnology. Antibodies against phospho-ERK1/2 (pThr^202^/pTyr^204^), phospho-PKB/Akt (pThr^308^), phospho-PKB/Akt (pSer^473^) were purchased from Cell Signaling Technology. The antibody against PKB/Akt was made in house. Secondary HRP (horseradish peroxidase)-conjugated antibodies (Bio-Rad Laboratories) and Alexa Fluor® 680/Alexa Fluor® 800-conjugated antibodies (Invitrogen) were used for ECL and Odyssey imagining respectively, according to the manufacturers’ instructions. LY294002 (final concentration 10 μM; SelleckChem), Akt1/2 inhibitor VIII (Akti; final concentration 10 μM; Santa Cruz Biotechnology), nocodazole (final concentration 250 ng/ml) and PD325901 {MEKi [MAPK (mitogen-activated protein kinase)/ERK kinase inhibitor]; final concentration 1 μM; SelleckChem} were dissolved in DMSO. 4OHT (4-hydroxytamoxifen; final concentration 500 nM; Sigma–Aldrich) was dissolved in 96% ethanol. Thymidine (final concentration 2.5 mM; Sigma–Aldrich) was dissolved in water.

### Plasmids, transfections and lentiviral transductions

pFakeEntry2Dummy was a kind gift from Dr. Holger Rehman (UMC Utrecht, The Netherlands) and constructed by transferring an attB flanked multiple cloning site into pDONOR201 (Invitrogen) using Gateway BP clonase (Invitrogen). Full-length human CTDSP2 (NM_005730.3) was amplified from HEK-293T cDNA and cloned using BamHI/EcoRI into pFakeEntry2Dummy and sequence-verified. The D98E/D101N mutant of CTDSP2 (CTDSP2^pd^), which lacks phosphatase activity [7], was generated following the Stratagene QuikChange® protocol. Previously described [20] p53^R175H^ was PCR-amplified with restriction sites and cloned using BamHI/NotI into pFakeEntry2Dummy. Plasmid pENTR4-V5-LUC (Addgene plasmid 19135) and pENTR4-GFP-C1 (Addgene plasmid 17396) have been described previously [21]. Using Gateway LR clonase (Invitrogen), GFP, V5-Luc, CTDSP2 and CTDSP2^pd^ were transferred into pInducer20 (Addgene plasmid 44012) [22]. p53^R175H^ was transferred into pLenti CMVTRE3G Puro DEST (Addgene plasmid 27565), a gift from Dr. Eric Campeau (University of Massachusetts Medical School, USA). Protein expression was induced using sterile filtered doxycycline (Sigma–Aldrich) at a final concentration of 1 μg/ml for 24 h unless stated otherwise. siRNA SmartPOOLs against hE2F7 (where h denotes human), hTp53, hCDKN1A (p21^Cip1/Waf1^) and CTDSP2 were all purchased from Fisher–Dharmacon and reverse-transfected using Hyperfect (Qiagen) with an RNA/Hyperfect ratio of 40 pmol:5 μl. Cell lines expressing different GFP-tagged FOXO isoforms were established by transfecting UTR [23] cells with pBIOPSF-DEST (modified from [24] to Gateway destination vector by F. Zwartkruis) containing full-length hFOXO1 (NM_002015.3), hFOXO3 (NM_001455.3) or hFOXO4 (NM_005938.3) using ExtremeGene (Roche) at a DNA/ExtremeGene ratio of 1:3 (w/v). Polyclonal lines were selected using 200 μg/ml Zeocin (Invitrogen) and monoclonal lines A13, B13 and C13 expressing comparable levels of tetracycline-inducible GFP–hFOXO1, GFP–hFOXO3 and GFP–hFOXO4 respectively, were established from these. Lentiviral particles were generated in HEK-293T cells as described previously [25]. Transient transfection of HEK-293T cells was mediated by MaxPEI (Polysciences) with a DNA/PEI (polyethyleneimine) ratio of 1:3 (v/v). Established polyclonal cell lines were selected for three weeks using 400–600 μg/ml G418 (Invitrogen). For luciferase experiments, a 479 bp genomic fragment overlapping the 5′-UTR of CTDSP2 was cloned using XhoI/HindIII into pGL3-Basic (Promega). A FOXO-binding-deficient promoter construct CTDSP2-mut was generated following the Strategene QuikChange® protocol, thereby changing TTGTTT to TTCTTT which efficiently reduces FOXO binding.

### Cell cycle profiling and viability assays

S-phase cells were detected using an FITC/BrdU (bromodeoxyuridine) staining kit (BD Biosciences) according to the manufacturer's instructions. For all cell lines used, BrdU was incubated for 30 min at 37°C. Alternatively, DNA content was measured using PI (propidium iodide; Sigma–Aldrich) staining as described previously [3]. Cell viability was measured by the ability of live cells to exclude PI as described in [3]. For all samples, 10000 cells were counted using a FACSCalibur instrument (BD Biosciences) and analysed using the provided software. For both BrdU-stained and PI-stained DNA content measurements, multi-cell-aggregates as well as sub-G_1_ cells were discarded for the analysis and G_0_/G_1_, S (when applicable) and G_2_/M populations were expressed as the percentage of total live cells (without sub-G_1_). FACS data are presented as means±S.D. for three biological experiments.

### RNA extraction, qPCR (quantitative PCR) and Western blotting

RNA was extracted using an RNeasy kit (Qiagen). cDNA was synthesized using iScript kit (Bio-Rad Laboratories) with 0.5 μg of RNA input. qPCR was performed using FastStart SYBR Green mix (Roche) using a CFX96 Real-Time Detection System (Bio-Rad Laboratories) and analysed using the software provided by the manufacturer. Expression levels were normalized to hTUBA1A or mHMBS (mouse hydroxymethylbilane synthase) [PBGD (porphobilinogen deaminase)] unless stated otherwise. qPCR data are presented as mean±S.D. (technical and biological) for three biological replicates. For each primer set, the average *C*_T_ value of the control samples was used to calculate fold change for all wells. Means±S.D. (technical) were calculated per sample measured, and S.D. propagated from normalization gene measurements to target gene measurements. S.D. calculations for biological replicates was performed according to equation 5.38 in [26]. Western blotting was performed according to standard laboratory protocols. Briefly, cells were lysed directly in Leammli buffer, boiled for 3 min and separated on denaturing SDS/PAGE gels. Proteins were transferred on to PVDF membrane, blocked with 2% BSA in TBS and strained with desired antibodies.

### ChIP

Anti-ERα (oestrogen receptor α) (M-20) and anti-FOXO3 (H-144) were purchased from Santa Cruz Biotechnology. Rabbit anti-GFP was a gift from Professor Dr G. Kops (UMC Utrecht, Utrecht, The Netherlands). The ChIP protocol has been described previously for DLD1/DL23 cells [27]. A13/B13/C13 cells were treated for 16 h with doxycycline and for 30 min with Akti before cross-linking, after which we followed the previously described protocol [27].

### *In vitro* kinase assays and GST–RBD (Ras-binding domain) pull-downs

Active CDK complexes precipitated from cells were treated with doxycycline for 24 h, washed twice with ice-cold PBS and lysed in 50 mM Tris/HCl (pH 7.5), 1% NP-40, 10% glycerol, 100 mM NaCl and protease/phosphatase inhibitors. Lysates were collected and cleared by centrifugation. Supernatants were incubated for 2 h with antibody-pre-coupled Protein A beads (Sigma–Aldrich), washed three times with lysis buffer and twice with kinase buffer (25 mM Tris/HCl, pH 7.5, 10 mM MgCl_2_ and 1 mM DTT). Beads were incubated for 30 min at 30°C in kinase buffer supplemented with 100 μM ATP, 10 μCi of γATP and 1 μg/reaction of recombinant H1 (Millipore) or 0.5 μg/reaction of recombinant GST–Rb (Sigma–Aldrich). Proteins were separated by SDS/PAGE and gels were briefly fixed in 30% methanol/10% acetic acid and wrapped in Saran Wrap (clingfilm) before X-film exposure. GST–Raf1–RBD pull-downs were performed as described previously [28].

### Luciferase assays

U2OS cells were transfected with 500 ng of pCDNA3 (EV; Invitrogen) or pCDNA3-HA-FOXO3.A3, 500 ng of CTDSP2/CTDSP2-mut and 50 ng of CMV (cytomegalovirus)–*Renilla* and 48 h post-transfection luciferase and *Renilla* expression was measured using a Dual-Luciferase Reporter Assay System (Promega) in a MicroLumat Plus LB 96V (Berthold Technologies), according to the manufacturers’ instructions. Luciferase counts were corrected for input using *Renilla* counts.

## RESULTS

### Defining a limited FOXO target gene signature reveals CTDSP2 as a FOXO target gene

**Figure 1:**
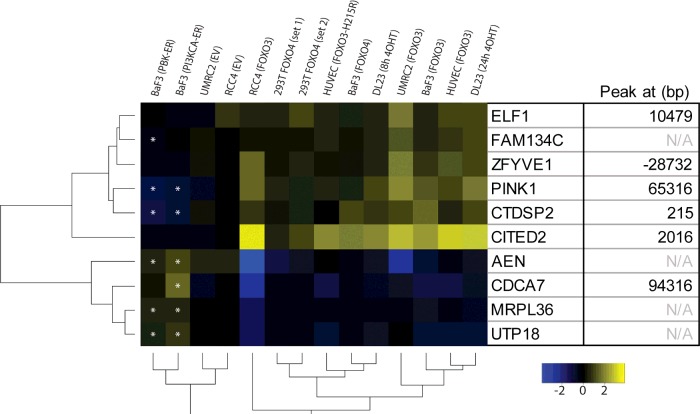
A common set of FOXO-regulated genes in human and mouse cell lines overexpressing FOXO3 or FOXO4 FOXO3 and FOXO4 induced changes in HUVECs and UMRC2, RCC4, BaF3, DLD1-derived DL23 [19] and HEK-293T cells were compared. Depicted are genes statistically significantly (*P*<0.05) regulated in relevant datasets (see the Materials and methods section for details). Asterisks indicate genes that are statistically significantly (ANOVA, *P*<0.05) regulated by PI3K or PKB/Akt activation, in opposing direction compared with FOXO3/4 activation. The right-hand side shows the distance of the closest FOXO3 peak in DL23 cells deduced from [27].

Active FOXO proteins affect a number of cellular processes (reviewed in [2]). To identify genes transcriptionally controlled by FOXO proteins that convey the regulation of these processes, different studies including the present one have generated microarray datasets [3–6]. These studies identified numerous FOXO target genes which, when analysed under different conditions, are shown to be regulated only in a particular context or cell type. To identify a set of FOXO3- and FOXO4-regulated genes that is common to at least a limited number of cell types, we merged microarray datasets of FOXO3- or FOXO4-induced expression changes in HUVECs (human umbilical vein endothelial cells), UMRC2, RCC4, BaF3, DLD1-derived DL23 [19] and HEK-293T cells (see the Supplementary methods for details). We found ten genes that are regulated in all datasets and focused on genes that are increased upon FOXO activation, because we have recently published that FOXO activation correlates best with increased expression of genes [27]. Of the six genes found to be statistically significantly (*P*<0.05) up-regulated in all datasets, *CITED2* and *PINK1* have been previously described to be FOXO target genes [29,30]. Besides these, *CTDSP2* is the most strongly regulated gene in these datasets, but has not been described as a FOXO target gene to date. Interestingly, CTDSP2 expression is decreased significantly (ANOVA, *P*<0.05) in response to activation of PI3K or PKB/Akt (Figure 1; two rightmost columns), suggesting that CTDSP2 expression is highly sensitive to FOXO activity.

### CTDSP2 is regulated by exogenous and endogenous FOXO proteins

**Figure 2:**
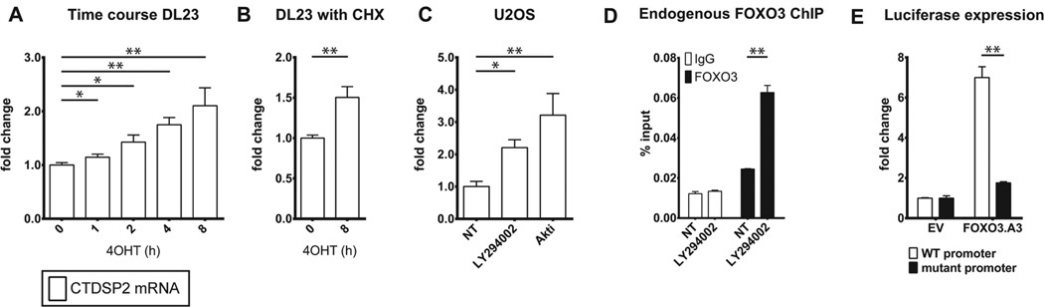
CTDSP2 expression is regulated by FOXO proteins (**A**) *CTDSP2* mRNA was measured by qPCR in DL23 cells after FOXO3 was activated by 4OHT for the indicated times or (**B**) for 8 h in the presence of cycloheximide (CHX). (**C**) U2OS cells were treated for 16 h with LY294002 or Akti to inhibit PI3K and PKB/Akt and thereby activate endogenous FOXO proteins. *CTDSP2* mRNA was measured by qPCR. Either treatment induces CTDSP2 expression significantly. (**D**) ChIP–qPCR-determined percentage input of the *CTDSP2* promoter precipitated by endogenous FOXO3 (black bars) or non-specific IgG (white bars). Nuclear localization of endogenous FOXO3 was induced using LY294002 and precipitated using anti-FOXO3 antibody. (**E**) U2OS cells were transfected as indicated in the Materials and methods section. FOXO3.A3 induces luciferase expression driven by a 479 bp fragment overlapping the 5′-UTR of human CTDSP2 (white bars), which is decreased if the FOXO-binding sites are mutated (black bars). WT, wild-type. Results are means±S.D. of three biological replicates or representative examples in the case of ChIP. Student's *t* tests: *P<0.05, **P<0.005; indication absent, *P*>0.05. NT, not treated.

To establish the kinetics of CTDSP2 regulation, we measured *CTDSP2* mRNA levels in response to activation of FOXO3.A3-ER in DL23 cells. We observed a rapid increase in *CTDSP2* mRNA levels after addition of 4OHT (Figure 2A), even in the presence of the protein translation inhibitor cycloheximide (Figure 2B), both strong indications that FOXO3 can directly control CTDSP2 expression. Furthermore, in DLD1, U2OS, RPE and NIH/3T3 cells, expression of *CTDSP2* mRNA is increased in response to inhibitors of PI3K or PKB/Akt (Figure 2C and Supplementary Figure S1A), which are known to control the activity of endogenous FOXO proteins [31]. Interestingly, CTDSP2 regulation is not restricted to FOXO3, as ectopic expression of GFP–FOXO1, GFP–FOXO3 or GFP–FOXO4 also elevates *CTDSP2* mRNA levels in U2OS cells (Supplementary Figure S1B). Lastly, shRNA-mediated knockdown of FOXO1 and FOXO3 in U2OS cells decreases the basal and PKB/Akt-inhibition-induced increase in *CTDSP2* mRNA (Supplementary Figure S1C), showing that endogenous FOXO1 and FOXO3 are involved in the control of CTDSP2 expression levels.

In line with direct regulation of CTDSP2 by FOXO proteins, we have identified peaks in the promoter of CTDSP2 in previously published ChIP-seq data for DL23 cells [27] (Supplementary Figure S2A). We have confirmed these findings for FOXO3 in DLD1 (Figure 2D) and DL23 cells (Supplementary Figure S2B), and for FOXO1, FOXO3 and FOXO4 in U2OS cells (Supplementary Figure S2C). Furthermore, a 400 bp fragment overlapping with the *CTDSP2* 5′-UTR, is strongly activated by expression of FOXO3.A3, which depends on the presence of the two binding sites (Figure 2E). Taken together, these results show that CTDSP2 is a genuine and direct target of FOXO1, FOXO3 and FOXO4 and that FOXO proteins have a significant impact on the expression levels of CTDSP2.

### CTDSP2 expression decreases the number of cells in S-phase

**Figure 3:**
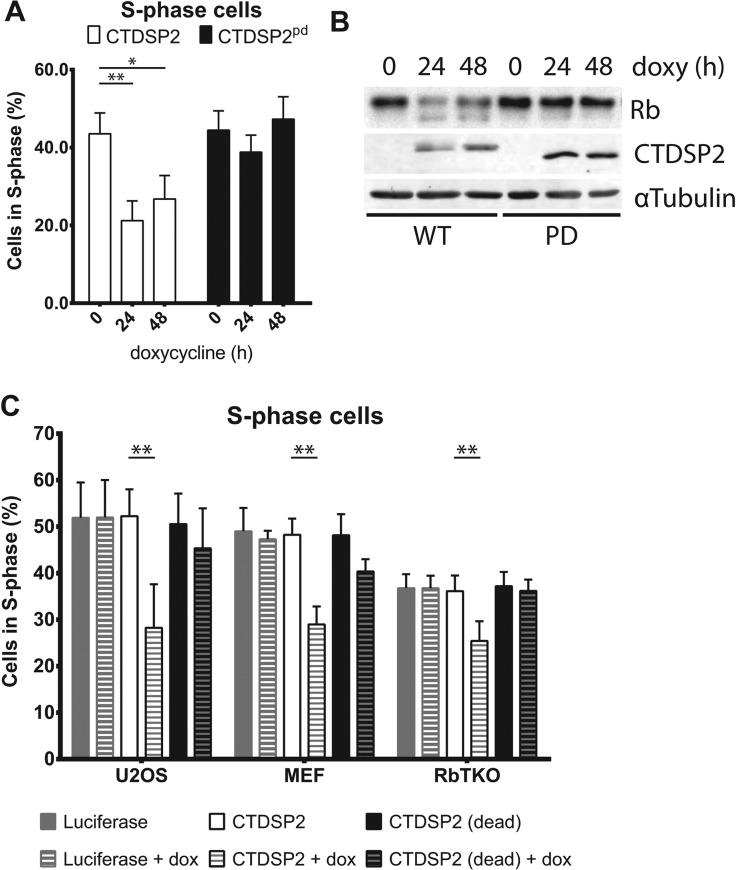
CTDSP2 expression decreases cell cycle progression (**A**) Expression of CTDSP2 and phosphatase-dead CTDSP2^pd^ in U2OS cells was induced for the indicated time. The percentage of BrdU-positive cells is decreased upon expression of CTDSP2 (white bars), but not phosphatase-dead CTDSP2^pd^ (black bars). (**B**) As (**A**) CTDSP2 (WT, wild-type) and phosphatase-dead CTDSP2^pd^ (PD) expression was induced for the indicated time, which results in decreased phosphorylation of pocket protein Rb (downshift of total Rb), as observed previously in [16,17]. α-Tubulin was used a loading control. (**C**) Luciferase, CTDSP2 or phosphatase-dead CTDSP2^pd^ expression was induced for 0 or 24 h (+dox) in U2OS, wild-type MEFs and MEFs lacking all pocket proteins before BrdU incorporation was measured. All cell lines arrest in response to CTDSP2 expression, showing that Rb is not required for cell cycle regulation by CTDSP2, unlike suggested previously in [16,17]. Results are means±S.D. of three biological replicates or representative examples in the case of Western blots. Student's *t* tests: *P<0.05, **P<0.005; indication absent, *P*>0.05. dox/doxy, doxycycline.

To study the effects of elevated CTDSP2 expression in isolation, we used a doxycycline-regulated expression system to ectopically express CTDSP2. Expression of CTDSP1, CTDSP2 or CTDSPL has been described previously to induce cell cycle arrest [16,17]. To negate overexpression artefacts, we compared expression of wild-type CTDSP2 with a previously described phosphatase-dead mutant, CTDSP2^pd^ [7]. We measured BrdU incorporation into U2OS, RPE, DLD1 and NIH/3T3 cells after doxycycline-induced expression of CTDSP2 or phosphatase-dead CTDSP2^pd^. In line with previous reports [16,17], U2OS and RPE cells indeed showed a phosphatase-activity-dependent reduction in the number of cells in S-phase (U2OS shown in Figure 3A; results not shown for RPE), which is not due to differences in expression levels of CTDSP2 and phosphatase-dead CTDSP2^pd^ (Supplementary Figure S3A). The reduction in BrdU-positive cells results from an arrest in G_1_ or G_2_ (in U2OS), as well as cell death (Supplementary Figure S3B).

Similar to our observations (Figure 3B), it has been shown that expression of CTDSPL alone [16] or in combination with CTDSP1 and CTDSP2 [17], results in decreased phosphorylation of Rb, which was suggested to be the cause of the decreased cell cycle progression. However, decreased phosphorylation of Rb can be both cause and consequence of reduced cell cycle progression. Thus we analysed the effect of CTDSP2 or phosphatase-dead CTDSP2^pd^ expression on cell cycle progression in wild-type MEFs and MEFs that lack any of the three pocket proteins, referred to as RbTKO MEFs [18]. Surprisingly, RbTKO MEFs show a reduction in BrdU-positive cells in response to CTDSP2 expression (Figure 3C), albeit to a lesser extent (29.2±12.4% reduction) than in wild-type MEFs (40.0±6.7% reduction) or U2OS cells (46.8±11.4% reduction). Thus CTDSP2 expression arrests cells, but this does not critically depend on Rb or any of the other pocket proteins.

### Up-regulation of p21^Cip1/Waf1^ mediates part of the CTDSP2-induced cell cycle arrest

**Figure 4:**
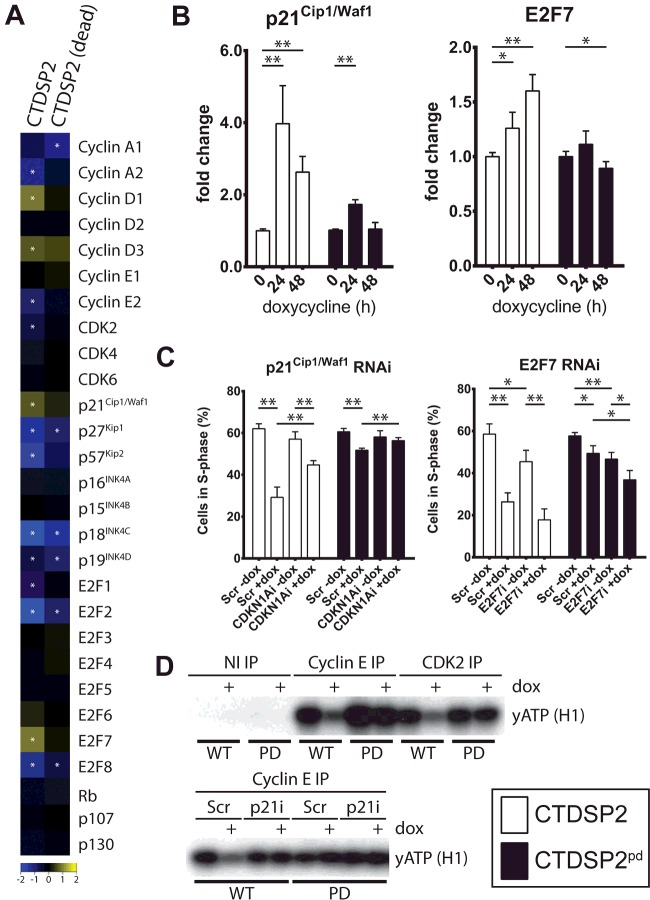
Cell cycle regulation by CTDSP2 involves p21^Cip1/Waf1^ (**A**) RNA was extracted from parental and U2OS cells expressing CTDSP2 or phosphatase-dead CTDSP2^pd^ for 48 h. Heatmap of microarray data showing expression of S-phase entry regulating genes (reviewed in [32]). Asterisk indicates statistically significant (ANOVA; *P*<0.05) regulation. Genes of interest that are specifically regulated by CTDSP2: cyclin E2 (1.77-fold down), p21^Cip1/Waf1^ (1.64-fold up), E2F1 (1.74-fold down) and E2F7 (1.88-fold up). (**B**) Expression of CTDSP2 and phosphatase-dead CTDSP2^pd^ was induced in U2OS cells for the indicated time and expression of the indicated gene was measured by qPCR. Both p21^Cip1/Waf1^ and E2F7 are increased upon CTDSP2 expression. (**C**) Either p21^Cip1/Waf1^ (+CDKN1Ai) or E2F7 (+E2F7i) was depleted using RNAi in U2OS cells expressing CTDSP2 or phosphatase-dead CTDSP2^pd^ for 0 (−dox) or 24 h (+dox). A non-targeting smartPOOL (+Scr) was used as a control. BrdU incorporation was measured after 0 (−dox) or 24 h expression of CTDSP2 or phosphatase-dead CTDSP2^pd^ (+dox). Depletion of p21^Cip1/Waf1^ (p21i) mitigates the ability of CTDSP2 to control cell cycle progression. (**D**) CDK2 and cyclin E-bound CDK were precipitated from U2OS cells expressing CTDSP2 (WT, wild-type) or phosphatase-dead CTDSP2^pd^ (PD) for 0 or 24 h (+). Kinase activity was measured as described in the Materials and methods section; more signal indicates more activity. CTDSP2 expression decreases both total and cyclin E-bound CDK2 activity (top panel) and depletion of p21^Cip1/Waf1^ restores cyclin E-bound CDK2 activity to its original level (bottom panel). Results are means±S.D. of three biological replicates or representative examples in the case of Western blots. Student's *t* tests: *P<0.05, **P<0.005; indication absent, *P*>0.05. dox, doxycycline.

Because Rb is not required for the CTDSP2-induced cell cycle arrest, we wanted to determine which genes are involved in the observed CTDSP2-induced cell cycle arrest. We collected gene expression profiles of CTDSP2- and phosphatase-dead CTDSP2^pd^-expressing U2OS cells and focused on the main regulators of the G_1_-to-S transition (reviewed in [32]). Interestingly, we observe that both the CDK inhibitor p21^Cip1/Waf1^ and E2F7 are statistically significantly up-regulated in CTDSP2, but not phosphatase-dead CTDSP2^pd^-expressing cells (Figures 4A and 4B, and Supplementary Figure S4A). Furthermore, E2F1 and cyclin E2 are down-regulated in a phosphatase-activity-dependent manner, in agreement with reduced S-phase entry (Figure 4A and Supplementary Figure S4B). Surprisingly, Cyclin D1 and Cyclin D3 are up-regulated in CTDSP2-expressing cells (Figure 4A and Supplementary Figure S4B), indicating that mitogenic signalling itself is not impaired.

E2F1 and (CDK2-bound) cyclin E2 are major constituents of the feedforward loop important during S-phase commitment (reviewed in [32]). Both reduced Rb phosphorylation and down-regulation of E2F1 potentially result in the observed reduction in cyclin E2 expression. Rb is phosphorylated and inactivated by cyclin D–CDK4/6 and cyclin E–CDK2, which can be inhibited by p21^Cip1/Waf1^, whereas E2F7 regulates E2F1 expression directly [33], thus making both proteins likely candidates to mediate the effects on cell cycle progression induced by CTDSP2 expression. We determined the relative contribution of p21^Cip1/Waf1^ and E2F7 up-regulation to the arrest induced by CTDSP2; RNAi-mediated reduction of either E2F7 or p21^Cip1/Waf1^ (Supplementary Figure S5A and S5B respectively) shows that p21^Cip1/Waf1^ plays a significant role in CTDSP2-induced cell cycle arrest (Figure 4C, left), whereas E2F7 does not play a significant role in this (Figure 4C, right). However, p21^Cip1/Waf1^ knockdown does not restore E2F1 expression to its original level (Supplementary Figure S5C).

The effect of p21^Cip1/Waf1^ on cell cycle progression is generally attributed to its ability to inhibit CDKs (reviewed in [34]) and the most relevant in the present study are CDK4/6 and CDK2 bound to cyclin E family members (reviewed in [32]). Indeed, the activity of total CDK2, cyclin E-bound CDK2 and CDK6 are greatly reduced by CTDSP2 expression (Figure 4D and Supplementary Figure S5E), which is not the result of reduced kinase input (Supplementary Figures S5D and S5E). Furthermore, we confirm that the reduced activity of cyclin E-bound CDK2 results from p21^Cip1/Waf1^ up-regulation, as knockdown of p21^Cip1/Waf1^ restores cyclin E-bound CDK2 activity to its original level (Figure 4D).

Taken together, these results show that CTDSP2 expression results in elevated expression of p21^Cip1/Waf1^, which inhibits cyclin–CDK activity and thereby reduces cell cycle progression.

### CTDSP2 regulates p21^Cip1/Waf1^ through growth factor signalling

**Figure 5:**
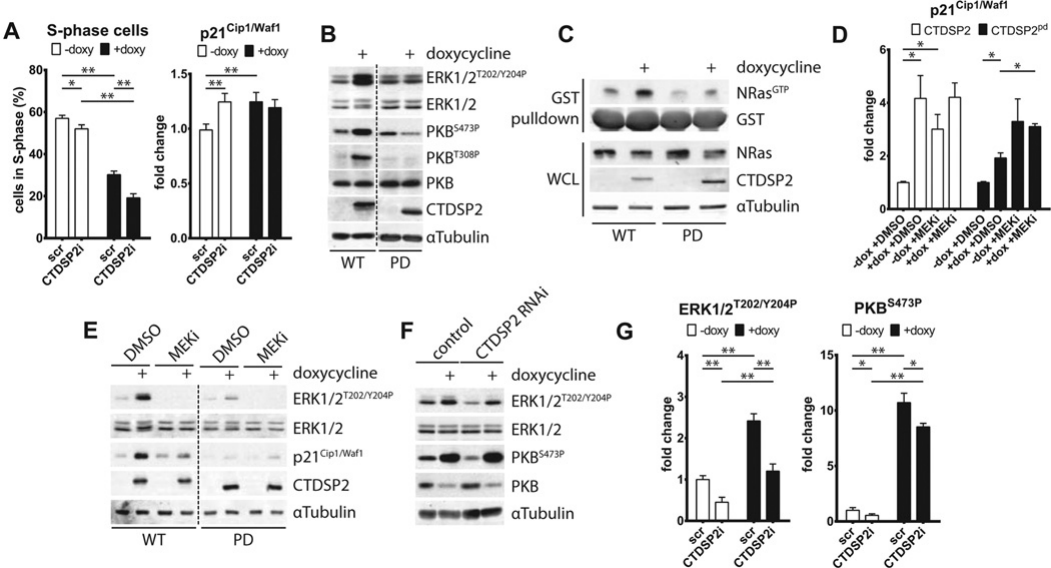
CTDSP2 regulates p21^Cip1/Waf1^ through growth factor signalling (**A**) CTDSP2 was depleted (CTDSP2i) using RNAi in U2OS cells expressing FOXO3.A3 for 0 (white bars) or 24 (black bars) h and BrdU incorporation (left panel) or p21^Cip1/Waf1^ mRNA expression (right panel) was measured. A non-targeting smartPOOL (scr) was used as a control. Both CTDSP2 depletion and FOXO3.A3 reduce S-phase entry and increases p21^Cip1/Waf1^ mRNA expression. CTDSP2 depletion exacerbates FOXO3.A3-induced cell cycle arrest and diminished FOXO3.A3-induced p21^Cip1/Waf1^ mRNA up-regulation. (**B**) CTDSP2 (WT, wild-type) and phosphatase-dead CTDSP2^pd^ (PD) expression was induced in U2OS cells for 0 or 24 h (+) and total protein was extracted. CTDSP2 expression induces phosphorylation of both ERK and PKB/Akt, indicating increased activity of either kinase. α-Tubulin was used a loading control. (**C**) As (**B**), but GTP-loaded Ras was precipitated using GST–Raf–RBD. Ras becomes activated upon expression of CTDSP2. WCL, whole-cell lysate. (**D**) As (**B**), but cells were treated for 24 h without (−dox) or with (+dox) doxycycline together with DMSO (+DMSO) or MEKi PD325901 (+MEKi). CTDSP2 does not induce p21^Cip1/Waf1^ mRNA to the same extent in the presence of MEKi. (**E**) As (**D**), but total protein was extracted. Western blots confirm decreased expression of p21^Cip1/Waf1^ in the presence of MEKi. (**F**) As (**A**), but total protein was extracted. CTDSP2 depletion decreases basal PKB/Akt and ERK phosphorylation, as well as FOXO3.A3-induced ERK and PKB/Akt phosphorylation. α-Tubulin was used a loading control. (**G**) Densitometric quantification of three experiments, including the one shown in (**F**). Phospho-ERK was corrected for total ERK and phospho-PKB/Akt was corrected for total PKB/Akt. CTDSP2 depletion statistically significantly reduces FOXO3.A3-induced phosphorylation of ERK and PKB/Akt. Results are means±S.D. of three biological replicates or representative examples in the case of Western blots. Student's *t* tests: *P<0.05, **P<0.005; indication absent, *P*>0.05.

Surprisingly, CTDSP2 knockdown in U2OS cells results in reduced cell cycle progression and increased p21^Cip1/Waf1^ expression (Figure 5A). In addition, in CTDSP2-depleted cells, the FOXO3.A3 expression-induced cell cycle arrest is exacerbated, whereas p21^Cip1/Waf1^ is no longer increased (Figure 5A). This prompted us to investigate further the mechanism underlying p21^Cip1/Waf1^ up-regulation upon CTDSP2 expression, as many pathways can affect the expression of p21^Cip1/Waf1^ (reviewed in [35,36]) and CTDSP2 is unlikely to directly regulate p21^Cip1/Waf1^ itself.

We identified several KEGG [37] pathways (as described in the Supplementary Methods) that are statistically significantly (χ^2^ test; *P*<0.05) affected by CTDSP2 expression, based on the changed expression of pathway components (pathways classified as Environmental Information Processing, Signal Transduction are summarized in Supplementary Figure S6). From a series of validation experiments, we have obtained evidence for the involvement of p53 and Ras–MAPK signalling in CTDSP2-induced p21^Cip1/Waf1^ expression.

The transcription factor p53 is well known for its regulation of p21^Cip1/Waf1^ and we observe that many p53-responsive genes are changed upon CTDSP2 expression (Supplementary Figures S7A and S7B). Furthermore, depletion of p53 (Supplementary Figure S7C) has a strong effect on basal expression of p21^Cip1/Waf1^ (Supplementary Figure S7D), similar to the expression of dominant-negative p53^R175H^ [20] (Supplementary Figure S7G). In addition to p53, other signalling cascades control the expression of p21^Cip1/Waf1^, as we still observe induction of p21^Cip1/Waf1^ upon CTDSP2 expression, albeit to a lesser extent (Supplementary Figures S7D, S7F and S7G). Furthermore, p53 depletion restored cell cycle progression to a lesser extent than p21^Cip1/Waf1^ depletion (Supplementary Figure S7E). Indeed, we have strong indications that activation of the Ras–Raf–MEK–ERK pathway is responsible for increased p21^Cip1/Waf1^ expression. First, upon CTDSP2 expression, we observed strong induction of ERK phosphorylation (Figure 5B). The increase in phosphorylation of ERK seems to result from activation of Ras, since RasGTP loading is increased by expression of CTDSP2 (Figure 5C). Confirming our observations on p53, Ras activation has been shown to result in p53-dependent and p53-independent increases in p21^Cip1/Waf1^ (reviewed in [36]). Interestingly, CTDSP2 is no longer able to increase p21^Cip1/Waf1^ to the same extent in the presence of an inhibitor of MEK (Figures 5D and 5E), which is critical in converting Ras activity into ERK phosphorylation.

Interesting in this context is that FOXO activation has been shown previously to activate growth factor signalling (reviewed in [38]). First, it has been described that FOXO activation results in increased Ras–Raf–MEK–ERK activity through increased expression of, e.g., HER3 (human epidermal growth factor receptor 3) [39]. Secondly, FOXO activation results in increased PI3K–PKB/Akt activity through regulation of many different genes (reviewed in [38]). Interestingly, PI3K–PKB/Akt signalling is also strongly activated upon CTDSP2 expression (Figure 5B). We observed increased phosphorylation of ERK and PKB/Akt upon expression of FOXO3.A3 in U2OS cells (Figure 5F). In line with decreased cell cycle progression, basal phosphorylation of ERK and PKB/Akt, and FOXO3.A3-induced phosphorylation of ERK and PKB/Akt is statistically significantly (Student's *t* test; *P*<0.05) lower in CTDSP2-depleted cells (Figure 5G). Taken together, this suggests that FOXO-induced CTDSP2 plays a role in activating growth factor signalling, in particular the activity of the Ras–Raf–MEK–ERK pathway.

## DISCUSSION

FOXO3 and FOXO4 target gene expression has been studied extensively over the years, but the datasets have not been combined before. In the present study, we examined a short list of genes regulated in all included datasets, thus representing a set of genes regulated by FOXO3 and FOXO4 in different cell types. In line with the notion that FOXO3 binding correlates predominantly with increased expression of genes [27], most up-regulated genes have one or more FOXO3-binding sites within 100 kb of the TSS (Figure 1). Only two out of six up-regulated genes were reported previously to be regulated by FOXO proteins, i.e. *CITED2* and *PINK1* [29,30]. The remaining genes might be of interest for further investigation. For example, *ZFYVE1* encodes a phosphatidylinositol 3-phosphate-binding protein that has been proposed to be involved in autophagy [40], a process that has also been linked to FOXO proteins [6]. About *FAM134C* relatively little is known. Interestingly, it has been shown to be a target gene of Nrf1 [41], a transcription factor that is important for stress resistance and linked to FOXO proteins [42]. These proteins are of particular interest because both autophagy and redox-stress regulation are strongly implicated in aging (reviewed in [2,38]). Lastly, Elf1 is an Ets-related transcription factor important in embryonic haemopoiesis and angiogenesis, its expression in adults being restricted to the lymphoid system (reviewed in [43]). Interestingly, FOXO1-knockout mice are embryonic lethal due to defects in vascular development [44,45] and induced loss of individual or multiple FOXO proteins at a later age results in vascular abnormalities [46].

In the present study, we have focused on the regulation of CTDSP2 and the consequences of CTDSP2 expression. CTDSP2 has not been described as a FOXO target gene previously. We have shown that CTDSP2 is regulated in response to FOXO activation in all cell lines we tested. Also, we show that FOXO1, FOXO3 and FOXO4 bind to and activate the promoter of CTDSP2, confirming and extending previous results from our laboratory [27]. Lastly, we show that FOXO proteins are important regulators of CTDSP2 in the context of PKB/Akt signalling as shRNA-mediated reduction of FOXO1 and FOXO3 results in decreased PKB/Akt-inhibition-induced expression of CTDSP2. Thus CTDSP2 is a novel ubiquitously regulated and direct target of FOXO proteins. In agreement with previous results [16,17], we show that expression of CTDSP2 results in a decreased number of cells in S-phase and we show that this is dependent on its phosphatase activity. Interestingly, our data indicate that the Rb family members are dispensable for CTDSP2-induced cell cycle arrest in contrast with previous suggestions [16,17]. Instead, we find that p21^Cip1/Waf1^ plays an important role in the CTDSP2-induced cell cycle arrest by decreasing the activity of cyclin–CDK complexes. Depletion of p21^Cip1/Waf1^ attenuated the CTDSP2 inhibition of cell cycle progression and restored cyclin E-bound CDK2 activity.

The increase in expression of p21^Cip1/Waf1^ appears to arise in response to CTDSP2-induced activation of Ras, which in turn regulates p21^Cip1/Waf1^ expression through p53-dependent and p53-independent mechanisms (reviewed in [36]). Indeed, our data indicate that blocking p53 or ERK signalling impairs the ability of CTDSP2 to induce p21^Cip1/Waf1^. However, our observations also illustrate the complexity of p21^Cip1/Waf1^ regulation. Whereas blocking p53 signalling decreases both basal and CTDSP2-induced expression of p21^Cip1/Waf1^, both decreased and increased ERK activity can result in p21^Cip1/Waf1^ expression ([47] and reviewed in [36] respectively).

Our data indicate that CTDSP2 can regulate changes in the activity of many different pathways, some of which have been shown to be able to regulate p21^Cip1/Waf1^ (reviewed in [35]). For example, TNFα (tumour necrosis factor α)/NF-κB (nuclear factor κB) signalling can activate p21^Cip1/Waf1^ expression, and many genes of this pathway are up-regulated in response to CTDSP2 expression. However, we observe that p65 Ser^276^ phosphorylation is decreased upon CTDSP2 expression and that IKKα/β (inhibitor of NF-κB kinase α/β) inhibitor VII cannot block CTDSP2-induced up-regulation of p21^Cip1/Waf1^ mRNA (results not shown). Furthermore, TGFβ signalling can affect p21^Cip1/Waf1^ expression in collaboration with FOXO proteins (reviewed in [48]), and TGFβ has been reported to be activated by CTDSP2 [13]. However, we observed decreased phosphorylation of both activating SXS phosphorylation and inactivating linker phosphorylation, which is supported by decreased SMAD7 expression (results not shown). In addition, we show that PKB/Akt is strongly activated by CTDSP2, making it unlikely that FOXO activation is responsible for p21^Cip1/Waf1^ up-regulation. Interestingly, inhibition of PKB/Akt does affect p21^Cip1/Waf1^ protein stability, but not mRNA expression levels (results not shown), adding another layer of complexity to the regulation of p21^Cip1/Waf1^ abundance. In summary, we provide evidence for a new target gene of FOXO proteins, regulated in a broad set of cellular contexts. Furthermore, we confirm and refine previous findings with respect to the regulation of cell cycle progression by CTDSP2 and point to p21^Cip1/Waf1^ as a specific mediator of these effects. Surprisingly, endogenous CTDSP2 promotes cell cycle progression through the regulation of the activity of growth factor signalling, i.e. Ras and PKB/Akt. As such, it is interesting to note that CTDSP2 was originally identified as co-amplifying with CDK4 [49]. These data strongly suggest that CTDSP2 participates in FOXO-induced activation of Ras and PKB/Akt signalling, which is thought an important mechanism for energy homoeostasis and a major drawback for the use of PI3K–PKB/Akt inhibitors in cancer.

## Abbreviations

Akti: Akt inhibitor
BrdU: bromodeoxyuridine
CDK: cyclin-dependent kinase
CTDSP: C-terminal domain small phosphatase
CTDSPL: CTDSP-like
ERK: extracellular-signal-regulated kinase
FOXO: forkhead box O
h: human
HEK: human embryonic kidney
HUVEC: human umbilical vein endothelial cell
MAPK: mitogen-activated protein kinase
MEF: mouse embryonic fibroblast
MEK: MAPK/ERK kinase
MEKi: MEK inhibitor
NF-κB: nuclear factor κB
4OHT: 4-hydroxytamoxifen
PI: propidium iodide
PI3K: phosphoinositide 3-kinase
PKB: protein kinase B
qPCR: quantitative PCR
Rb: retinoblastoma
RBD: Ras-binding domain
RPE: retinal pigment epithelium
TGFβ: transforming growth factor β
TSS: transcriptional start site
TUBA: α-tubulin
